# Clinical and life style factors related to the nighttime blood pressure, nighttime dipping and their phenotypes in Korean hypertensive patients

**DOI:** 10.1186/s40885-023-00241-w

**Published:** 2023-08-01

**Authors:** Byung Sik Kim, Ju Han Kim, Wan Kim, Woo Shik Kim, Sungha Park, Sang Jae Lee, Jang Young Kim, Eun Mi Lee, Sang Hyun Ihm, Wook Bum Pyun, Jeong-Hun Shin, Jinho Shin

**Affiliations:** 1 Division of Cardiology, Department of Internal Medicine, Hanyang University Guri Hospital, Guri, South Korea; 2 Division of Cardiology, Department of Internal Medicine, Chonnam National University Hospital, Gwangju, South Korea; 3 Division of Cardiology, Department of Internal Medicine, Gwangju Veterans Hospital, Gwangju, South Korea; 4 Division of Cardiology, Department of Internal Medicine, Kyung Hee University Medical Center, Seoul, South Korea; 5 Division of Cardiology, Department of Internal Medicine, Severance Cardiovascular Hospital, Yonsei University, Seoul, South Korea; 6 Division of Cardiology, Department of Internal Medicine, Wonkwang University Hospital, Iksan, South Korea; 7 Division of Cardiology, Department of Internal Medicine, Wonju Severance Christian Hospital, Yonsei University Wonju College of Medicine, Wonju, South Korea; 8 Division of Cardiology, Department of Internal Medicine, Wonkwang University Hospital, Sanbon, South Korea; 9 Division of Cardiology, Department of Internal Medicine, Catholic University School of Medicine, Seoul, South Korea; 10 Division of Cardiology, Department of Internal Medicine, Ehwa Women’s University Seoul Hospital, Seoul, South Korea; 11 Division of Cardiology, Department of Internal Medicine, Hanyang University Medical Center, Hanyang University College of Medicine, 222, Wangsimni-Ro, Sungdong-Gu, Seoul, 04763 South Korea

**Keywords:** Blood Pressure Monitoring, Ambulatory, Blood pressure, Hypertension, Sleep, Antihypertensive Agents, Smoking, Aging

## Abstract

**Background:**

Non-dipping or reverse dipping patterns are known to be associated with adverse cardiovascular prognosis among the general population and clinical cohort. Few large sized studies have explored factors including sleep duration and sleep quality related to nighttime blood pressure (BP) and nocturnal dipping patterns.

**Methods:**

Among 5,360 patients enrolled  in Korean multicenter nationwide prospective Registry of ambulatory BP monitoring (KORABP), 981 subjects with complete data on sleep duration, sleep quality assessed using a 4-point Likert scale, and clinical variables were included in the analysis. Phenotypes of nighttime BP pattern were categorized as extreme dipper, dipper, non-dipper, and reverse dipper. Hypertension was defined as a 24-h ambulatory BPs were 130/80 mmHg or higher.

**Results:**

Among 981 subjects, 221 were normotensive, 359 were untreated hypertensive, and 401 were treated hypertensive. Age of the participants were 53.87 ± 14.02 years and 47.1% were female. In overall patients, sleep duration was 431.99 ± 107.61 min, and one to four points of sleep quality were observed in 15.5%, 30.0%, 30.4%, and 24.2%, respectively. Of the 760 hypertensive patients, extreme dipper, dipper, non-dipper, and reverse dipper were observed in 58 (7.63%), 277 (36.45%), 325 (42.76%), and 100 (13.16%), respectively. In multiple linear regression analysis, sleep duration (β = 0.0105, *p* < 0.001) and sleep quality (β = -0.8093, *p* < 0.001) were associated with nighttime systolic BP and sleep quality was associated with extent of nighttime systolic BP dipping (β = 0.7622, *p* < 0.001) in hypertensive patients. In addition, sleep quality showed positive association with dipper pattern (odds ratio [OR] = 1.16, 95% confidence interval [CI] = 1.03–1.30) and showed negative association with reverse dipper pattern (OR = 0.73, 95% CI = 0.62–0.86) in multiple logistic regression analyses.

**Conclusion:**

When adjusted covariates, less sleep duration and poor sleep quality were positively associated with nighttime systolic BP. Additionally, sleep quality was the independent associated factor for dipper and reverse dipper phenotypes. The study also found that male sex, low estimated glomerular filtration rate, high ambulatory BP, low office BP, and poor sleep quality were associated with blunted nighttime SBP dipping.

**Supplementary Information:**

The online version contains supplementary material available at 10.1186/s40885-023-00241-w.

## Introduction

Hypertension is the key risk factor for cardiovascular events and deaths globally [1]. Despite the progress in blood pressure (BP) management strategies, BP control rate remain an unmet challenge for public health [2–4]. Difficulties in controlling the BP can be explained by a several factors, including patient related factors, awareness related factors, drug related factors, and lifestyle related factors [5, 6]. Lack of awareness of white-coat and masked which are related to accurate BP monitoring is also important factor hindering optimal BP control [7–9]. This is caused by the patients measured their BP only at clinic, so the importance of out-of-office BP measurement through ambulatory BP monitoring (ABPM) or home BP monitoring is emphasized.

Another important residual risk in the management of hypertension is nocturnal hypertension, which can even be a risk factor in patients whose BP are well controlled in daytime. It is well known that nighttime BP and it’s dipping pattern are important for prognosis [8, 10]. However, background factors associated with nighttime BP and dipping patterns of nighttime BP are remain unclear. Previously, few studies have been reported that advanced age, sleep disorder, alcohol consumption, diabetes mellitus, chronic kidney disease, and obstructive sleep apnea might be related with nighttime BP [11–14]. Dipping patterns of nighttime BP are also related to several factors [15, 16]. Advanced age, obesity, diabetes mellitus, and cardiovascular or renal disease were reported as an associated factor for blunted nocturnal dipping, which are related to poor prognosis [17]. Another important factor related to nighttime BP and it’s dipping pattern are sleep pattern. Disturbance in the quantity or quality of sleep may contribute to the development of nocturnal hypertension [10, 18]. However, there are few studies to investigate the factors related to nighttime BP and nocturnal dipping patterns including sleep duration and sleep quality has been reported [19].

Even though nighttime BP and it’s dipping pattern is known to be important for the prediction for cardiovascular outcomes, the major guidelines have not introduced specific management recommendation for the control of the dipping pattern of nighttime blood pressure. One of the most important barriers to the research for nighttime BP pattern is the poor reproducibility [20]. How to define the sleep time is also an important issue in nighttime BP measurement, given its recognized influence on the dipping patterns of nighttime BP [21]. The aforementioned limitations regarding obtaining nighttime BP, especially during sleep-time BP, may be difficult to overcome in situations where the current "cuff-based" ABPM method is the only widely available method for assessing nighttime BP, even though it can easily disturb sleep [22].

To overcome this limitation, it is still important to investigate the factors related to nighttime BP and it’s dipping pattern because considerations of those factors could improve the reproducibility of nighttime BP parameters. In this study, using the data from Korean Registry for ABPM (KORABP) in which lifestyle factors such as regular physical exercise, smoking and alcohol habit, and questionnaire for subjective sleep quality during ABPM were included, the factors associated with nighttime BP and it’s dipping patterns were analyzed.

## Methods

### Study population

This study was conducted using data from KORABP registry, a multicenter observational registry that aims to determine the prognostic threshold of ambulatory BP for predicting clinical outcomes in Korean hypertensive patients referred to secondary or tertiary hospitals as detailed in the previous report [23]. Among data for 5,360 patients available in the second version of the KORABP database, 981 subjects (221 normotensives and, 760 hypertensives) with complete data for questionnaire, laboratory and clinical data were analyzed [24].

### Blood pressure measurements

Office BP was measured by physicians or nurses using an upper arm cuff oscillometric BP device (A&D, UA-767, Tokyo, Japan) after the participants had rested for 5 min the sitting position. Two measurements were taken at 1 min intervals, and the average was used to define office systolic BP (SBP) and diastolic BP (DBP).

ABPM was carried out with a validated automatic oscillometric method device that passed the Association for the Advancement of Medical Instrumentation (AAMI) or the European Society of Hypertension (ESH) validation protocol for 15–30 min intervals during the daytime and 30–60 min intervals during the nighttime as reported previously [25]. Raw data files of ABPM were defined as valid when at least 20 readings during daytime and at least 7 readings during nighttime were available, after omitting erroneous reading according to the following criteria: 1) SBP < 70 mmHg or > 250 mmHg, 2) DBP < 40 mmHg or > 150 mmHg, and 3) pulse pressure < 20 mmHg or > 150 mmHg [26].

### Definition of hypertension

Hypertension was defined when a patient was taking antihypertensive drug therapy or when 24-h BPs were 130/80 mmHg or higher. Hypertension by office BPs was defined as office BPs were 140/90 mmHg or higher [27].

### Assessment of physical activity, smoking and alcohol habits

The questionnaire used for Korean National Health and Nutrition Survey to examine physical activity, smoking and alcohol habits were used. Regular physical activity was defined as 3 or more structured exercise of moderate intensity or higher per week. Smoking was defined as current smoker and alcohol drinking was defined as current drinking at least one time per month. Alcohol intake at the day of ABPM was not collected [28].

### Assessment of sleep duration and quality

Sleep duration was defined by the time from the stating of sleep and the time of awakening by the patient’s diary. Sleep quality was assessed by the questions of “How was the sleep last night?” and the response was choosing one of four Likert scales, 1-point for hardly sleep, 2-points for poor sleep, 3-points for not so bad, and 4-points for very good.

### Definition of nighttime blood pressure and nocturnal dipping pattern

Nighttime SBP dipping was defined as decrease in SBP during sleep compared to that during daytime. Nighttime BP was defined by average BP during actual sleep time. Daytime was defined as the time interval from 8am to 9 pm. Average BP was calculated using the number of readings and the weighting for the measurement interval between readings. Twenty-four-hour BP was defined as nighttime BP x actual sleeping duration/24 + daytime BP x (24 minus actual sleep duration)/24. Nighttime BP by narrow fixed interval method was defined by average BP from midnight to 5AM and nighttime BP during deep sleep was defined by the average BP between the time of 2 h after sleep and the time of 1 h before awakening. We sub-classified the patients according to the percentage of nighttime SBP decline as follows: extreme dippers if the nighttime SBP reduction was 20% at least; dippers if the fall was 10% at least but < 20%; non-dippers if the fall was 0% at least but < 10%; and the reverse dippers if it was < 0% [29].

### Clinical data

Clinical data were obtained using a web-based electronic data capture system that included electronic case report forms from the KORABP registry database. The following characteristics were collected: age, gender, height, weight, and medical history including the diagnosis of hypertension, diabetes, dyslipidemia, myocardial infarction, coronary artery disease, and stroke, along with data on treatment for any of the above conditions. The following laboratory data were collected: fasting glucose level, total cholesterol, and creatinine. Dyslipidemia was defined from the records based on the diagnosis of dyslipidemia, the use of statins, or total cholesterol levels ≥ 240 mg/dL. Diabetes mellitus was defined based on past medical history, fasting blood glucose level was 126 mg/dl or higher or hemoglobin A1C was 6.5% or higher. Estimated glomerular filtration rate (eGFR) was calculated using the Chronic Kidney Disease Epidemiology Collaboration (CKD-EPI) formula.

### Statistical analyses

Data were expressed as mean ± standard deviation or number with percentage, as appropriate. Clinical characteristics were described according to the hypertension status and antihypertensive medication status. In addition, clinical characteristics according to the nocturnal dipping pattern in hypertensive patients were also described. The difference among groups in the continuous variables was tested with ANOVA and Scheffe post-hoc multiple comparison test. The difference of the distribution among the group in the categorical variables was tested using the Chi-square test. The factors associated with nighttime SBP and the extent of nighttime SBP dipping were explored using multiple linear regression analyses including independent variables such as age, sex, regular physical activity which was defined as 3 or more moderate intensity exercise per week, body mass index (BMI), current smoking, alcohol drinking, antihypertensive medication status, medical histories of diabetes mellitus and dyslipidemia, medical history of cardiovascular disease such as coronary artery disease, stroke, and heart failure, total cholesterol, eGFR, 24-h mean SBP, office SBP, sleep duration, and sleep quality. The factors associated with nighttime dipping less than 10% and each nocturnal dipping pattern, i.e., dipper, non-dipper, reverse dipper, and extreme dipper were explored using generalized linear models including the relevant covariates such as, age, sex, regular physical activity, BMI, current smoking, alcohol drinking, antihypertensive medication status, medical histories of diabetes mellitus and dyslipidemia, medical history of cardiovascular disease such as coronary artery disease, stroke, and heart failure, eGFR, 24-h mean SBP, office SBP, sleep duration, and sleep quality. Analyses were also performed using different definitions of nighttime BP, i.e., mean BP without time weighting, mean BP with narrow fixed time interval method, and mean BP during deep sleep were described. McNemar’s test and Cochran’s Q test were used to compare the proportion of each dipping pattern between different definitions of nighttime BP. *P* value < 0.05 was considered as statistically significant. RStudio ver 1.1.414 and R ver 4.2.1 were used for the statistical analysis.

## Results

### Clinical characteristics

Among the 981 patients, 221 were normotensive, 359 were untreated hypertensive and, 401 were treated hypertensive. In overall patients, sleep duration was 431.99 ± 107.61 min, and one to four points of sleep quality were observed in 15.5%, 30.0%, 30.4%, and 24.2%, respectively. Clinical characteristics of the study patients according to the hypertension status and antihypertensive medication status were summarized in Table 1. There were no significant differences among the groups of normotensive, untreated hypertensive, and treated hypertensive, in terms of sex, regular physical activity, alcohol drinking, sleep duration and sleep quality. There were significant differences in the age, BMI, comorbidities, laboratory findings, office and ambulatory BPs. BPs and frequency of comorbidities were generally higher in hypertensive patients. In the nocturnal dipping pattern, the proportion of extreme dipper and non-dipper significantly differed between the groups, but there were no differences in the proportion of dipper and reverse dipper.

**Table 1:** Clinical characteristics according to the hypertension status and antihypertensive medication status

	Overall (*n* = 981)	Normotensive (*n* = 221)	Untreated hypertensive (*n* = 359)	Treated hypertensive (*n* = 401)	*p*-value
Age (years)	53.87 ± 14.02	53.3 ± 14.3^ab^	50.2 ± 14.0^a^	57.5 ± 13.0	< 0.001
Female (%)	462 (47.1)	111 (50.2)	159 (44.3)	192 (47.9)	0.349
Regular physical activity (%)	353 (36.0)	77 (34.8)	132 (36.8)	144 (35.9)	0.895
Body mass index, (kg/m^2^)	24.68 ± 3.30	24.0 ± 3.0^ab^	24.8 ± 3.1	25.0 ± 3.5	0.001
Obesity (%)^c^	412 (42.0)	75 (33.9)	156 (43.5)	181 (45.1)	0.02
Current smoking (%)	152 (15.5)	30 (13.6)	69 (19.2)	53 (13.2)	0.049
Alcohol drinking (%)	382 (38.9)	80 (36.2)	152 (42.3)	150 (37.4)	0.242
Comorbidities (%)					
Diabetes mellitus	199 (20.3)	29 (13.1)	54 (15.0)	116 (28.9)	< 0.001
Dyslipidemia	320 (32.6)	43 (19.5)	76 (21.2)	201 (50.1)	< 0.001
Cardiovascular diseases^d^	120 (12.2)	0 (0.0)	11 (3.1)	109 (27.2)	< 0.001
Myocardial infarction	25 (2.6)	0 (0.0)	1 (0.3)	24 (6.1)	< 0.001
Coronary artery disease	90 (9.2)	0 (0.0)	4 (1.1)	86 (21.4)	< 0.001
Stroke	27 (2.8)	0 (0.0)	6 (1.7)	21 (5.3)	< 0.001
Laboratory tests					
Fasting blood glucose (mg/dL)	105.43 ± 25.51	104.62 ± 26.0	102.89 ± 21.86^a^	108.03 ± 28.00	0.019
Total cholesterol (mg/dL)	189.75 ± 42.65	189.77 ± 42.38	196.83 ± 41.07^a^	183.39 ± 43.28	< 0.001
eGFR (ml/min/1.73m^2^)	83.85 ± 20.91	86.83 ± 17.91^a^	88.98 ± 20.42^a^	77.61 ± 21.32	< 0.001
eGFR < 60 (%)	83 ± 8.5	14 (6.3)	12 (3.3)	57 (14.2)	< 0.001
Office BP					
Office SBP (mmHg)	141.67 ± 21.51	122.48 ± 11.75^ab^	149.19 ± 18.83^a^	145.51 ± 21.71	< 0.001
Office DBP (mmHg)	88.59 ± 14.95	76.43 ± 8.62^ab^	94.16 ± 13.32^a^	90.30 ± 15.35	< 0.001
Heart rate (beats/min)	76.38 ± 13.91	74.23 ± 12.74^b^	78.21 ± 14.93^a^	75.74 ± 13.31	0.003
Ambulatory BP monitoring					
24-h mean SBP (mmHg)	133.56 ± 15.44	125.46 ± 13.09^ab^	136.99 ± 14.76	134.95 ± 15.69	< 0.001
24-h mean DBP (mmHg)	83.67 ± 10.87	78.57 ± 9.41^ab^	87.39 ± 11.19^a^	83.15 ± 10.09	< 0.001
Daytime mean SBP (mmHg)	137.37 ± 15.81	128.56 ± 13.55^ab^	141.00 ± 15.03	138.98 ± 15.87	< 0.001
Daytime mean DBP (mmHg)	86.49 ± 11.49	80.91 ± 10.00^ab^	90.52 ± 11.86^a^	85.96 ± 10.52	< 0.001
Nighttime mean SBP (mmHg)	125.62 ± 17.35	119.07 ± 14.58^ab^	128.56 ± 16.51	126.60 ± 18.56	< 0.001
Nighttime mean DBP (mmHg)	77.79 ± 11.31	73.88 ± 9.75^ab^	80.83 ± 11.46^a^	77.24 ± 11.24	< 0.001
Nighttime SBP dipping (%)	8.46 ± 7.89	7.24 ± 7.60^a^	8.75 ± 7.32	8.86 ± 8.47	0.034
Nocturnal dipping pattern (%)					
Extreme dipper	63 (6.4)	5 (2.3)	20 (5.6)	38 (9.5)	0.001
Dipper	349 (35.6)	72 (32.6)	130 (36.2)	147 (36.7)	0.567
Non-dipper	433 (44.1)	108 (48.9)	170 (47.4)	155 (38.7)	0.015
Reverse dipper	136 (13.9)	36 (16.3)	39 (10.9)	61 (15.2)	0.11
Sleep duration (min)	431.99 ± 107.61	436.62 ± 93.03	433.09 ± 96.84	428.46 ± 123.27	0.646
Sleep quality					0.899
4 (very good) (%)	237 (24.2)	57 (25.8)	80 (22.3)	100 (24.9)	
3 (good) (%)	298 (30.4)	64 (29.0)	109 (30.4)	125 (31.2)	
2 (bad) (%)	294 (30.0)	63 (28.5)	115 (32.0)	116 (28.9)	
1 (very bad) (%)	152 (15.5)	37 (16.7)	55 (15.3)	60 (15.0)	

Data are presented as *n* (%) or mean ± standard deviation, as appropriate. eGFR, estimated glomerular filtration rate; BP, blood pressure; SBP, systolic blood pressure; DBP, diastolic blood pressure

^a^Post hoc *p*: Statistically significant difference *p* < 0.05 compared to the Treated hypertensive

^b^Post hoc *p*: Statistically significant difference *p* < 0.05 compared to the Untreated hypertensive

^c^Obesity is defined as a body mass index of 25 or higher

^d^Cardiovascular diseases are defined as a composite of myocardial infarction, coronary artery disease, and stroke

### Comparison of characteristics according to the nocturnal dipping pattern in hypertensive patients

As shown in Table 2, among the group of nocturnal dipping patterns in hypertensive patients, there were significant differences in the age, sex, proportion of participants taking antihypertensive drugs, medical history of myocardial infarction and stroke, laboratory findings such as low eGFR (< 60 ml/min/1.73m2), and BPs such as 24-h mean SBP and DBP, daytime mean DBP, and nighttime SBP and DBP. There were significant differences in sleep quality between the groups, while there was no difference in sleep duration between the groups.

**Table 2:** Clinical characteristics according to the nocturnal dipping pattern in hypertensive patients

	Extreme dipper (*n* = 58)	Dipper (*n* = 277)	Non-dipper (*n* = 325)	Reverse dipper (*n* = 100)	*p*-value
Age (years)	51.02 ± 13.11^a^	52.95 ± 13.27^a^	54.22 ± 14.17	58.13 ± 14.71	0.004
Female (%)	17 (29.3)	114 (41.2)	168 (51.7)	52 (52.0)	0.002
Regular physical activity (%)	23 (39.7)	102 (36.8)	112 (34.5)	39 (39.0)	0.776
Body mass index, (kg/m^2^)	25.42 ± 3.04^a^	24.80 ± 3.18^a^	24.90 ± 3.40^a^	24.75 ± 3.81	0.613
Obesity (%)^d^	29 (50.0)	125 (45.1)	142 (43.7)	41 (41.0)	0.722
Current smoking (%)	14 (24.1)	50 (18.1)	49 (15.1)	9 (9.0)	0.056
Alcohol drinking (%)	30 (51.7)	116 (41.9)	124 (38.2)	32 (32.0)	0.077
Taking antihypertensive drugs (%)	38 (65.5)	147 (53.1)	155 (47.7)	61 (61.0)	0.02
Comorbidities (%)					
Diabetes mellitus	10 (17.2)	63 (22.7)	71 (21.8)	26 (26.0)	0.634
Dyslipidemia	20 (34.5)	103 (37.2)	111 (34.2)	43 (43.0)	0.431
Cardiovascular diseases^e^	9 (15.5)	49 (17.7)	42 (12.9)	20 (20.0)	0.251
Myocardial infarction	6 (10.3)	8 (2.9)	8 (2.5)	3 (3.1)	0.02
Coronary artery disease	8 (13.8)	39 (14.1)	33 (10.2)	10 (10.0)	0.431
Stroke	0 (0.0)	9 (3.3)	5 (1.5)	13 (13.1)	< 0.001
Laboratory tests					
Fasting blood glucose (mg/dL)	103.41 ± 21.45	104.74 ± 23.95	106.22 ± 27.22	107.22 ± 25.46	0.719
Total cholesterol (mg/dL)	194.41 ± 44.00	189.81 ± 43.10	191.22 ± 40.95	182.00 ± 46.37	0.225
eGFR (ml/min/1.73m^2^)	84.27 ± 16.43	83.38 ± 20.40	83.98 ± 20.78	77.88 ± 28.86	0.088
eGFR < 60 (%)	2 (3.4)	20 (7.2)	28 (8.6)	19 (19.0)	0.002
Office BP					
Office SBP (mmHg)	145.16 ± 16.69	147.27 ± 18.35	147.98 ± 20.91	146.01 ± 26.01	0.711
Office DBP (mmHg)	95.08 ± 13.93	92.62 ± 14.10	91.64 ± 14.10	90.59 ± 17.22	0.243
Heart rate (beats/min)	77.05 ± 13.12	76.09 ± 12.78	77.61 ± 14.68	76.79 ± 16.48	0.626
Ambulatory BP monitoring					
24-h mean SBP (mmHg)	130.71 ± 12.40^ab^	133.50 ± 14.08^ab^	137.69 ± 14.53	139.86 ± 20.00	< 0.001
24-h mean DBP (mmHg)	82.41 ± 10.18	84.23 ± 10.18	86.32 ± 11.05	85.48 ± 11.77	0.022
Daytime mean SBP (mmHg)	142.01 ± 13.59	139.88 ± 14.82	140.21 ± 14.92	138.02 ± 19.73	0.446
Daytime mean DBP (mmHg)	89.36 ± 11.80	88.66 ± 10.81	88.35 ± 11.57	85.11 ± 11.84	0.037
Nighttime mean SBP (mmHg)	108.63 ± 10.90^abc^	119.80 ± 12.94^ab^	132.35 ± 14.18^a^	144.20 ± 21.41	< 0.001
Nighttime mean DBP (mmHg)	68.65 ± 7.92^abc^	74.76 ± 9.44^ab^	82.01 ± 10.72^a^	86.44 ± 12.27	< 0.001
Nighttime SBP dipping (%)	23.49 ± 2.68^abc^	14.33 ± 2.68^ab^	5.57 ± 2.66^a^	-4.47 ± 3.48	< 0.001
Sleep duration (min)	433.33 ± 75.55	429.65 ± 123.19	433.71 ± 101.10	421.98 ± 127.04	0.825
Sleep quality					0.001
4 (very good) (%)	13 (22.4)	72 (26.0)	80 (24.6)	15 (15.0)	
3 (good) (%)	20 (34.5)	93 (33.6)	94 (28.9)	27 (27.0)	
2 (bad) (%)	14 (24.1)	92 (33.2)	90 (27.7)	35 (35.0)	
1 (very bad) (%)	11 (19.0)	20 (7.2)	61 (18.8)	23 (23.0)	

Data are presented as *n* (%) or mean ± standard deviation, as appropriate. eGFR, estimated glomerular filtration rate; BP, blood pressure; SBP, systolic blood pressure; DBP, diastolic blood pressure

^a^Post hoc *p*: Statistically significant difference *p* < 0.05 compared to the Reverse dipper

^b^Post hoc *p*: Statistically significant difference *p* < 0.05 compared to the Non-dipper

^c^Post hoc *p*: Statistically significant difference *p* < 0.05 compared to the Dipper

^d^Obesity is defined as a body mass index of 25 or higher

^e^Cardiovascular diseases are defined as a composite of myocardial infarction, coronary artery disease, and stroke

### Relationship between sleep duration and sleep quality with nighttime blood pressure and nighttime systolic blood pressure dipping

As shown in Table 3, sleep duration (β = 0.0105, *p* < 0.001) and sleep quality (β = -0.8093, *p* < 0.001) were significantly associated with nighttime SBP in multiple linear regression analysis. In addition, sleep quality was also significantly associated with extent of nighttime SBP dipping (β = 0.7622, *p* < 0.001), but sleep duration was not associated with extent of nighttime SBP dipping (β = -0.0036, *p* = 0.081) (Table 4). Age, sex, SBP, smoking status, and total cholesterol were also associated factors for nighttime SBP and extent of nighttime SBP dipping. Since there is a possibility that office SBP and 24-h mean SBP are not independent of each other, we excluded each from the model and analyzed them separately. Table S1 showed the association of other variables with nighttime SBP dipping was consistent in both models. However, in the analysis performed excluding 24-h SBP, office SBP did not show significant association with nighttime SBP dipping.

**Table 3:** Multiple linear regression analysis for the factor associated with nighttime systolic blood pressure in hypertensive patients

	β coefficient	Standard error	*p*-value
Age (per 1 year)	0.0441	0.0172	0.01
Female	-1.0248	0.473	0.03
Regular physical activity	0.2418	0.4323	0.576
Body mass index (per 1 kg/m2)	-0.0303	0.0612	0.62
Current smoking	-1.4245	0.612	0.02
Alcohol drinking	-0.836	0.4732	0.078
Taking antihypertensive drug	-0.6332	0.4483	0.158
Medical history of diabetes mellitus	0.9556	0.5223	0.068
Medical history of cardiovascular disease^a^	0.5548	0.6055	0.36
Total cholesterol (per 1 mg/dL)	-0.0157	0.0049	0.001
eGFR (per 1 ml/min/1.73m2)	-0.0197	0.0105	0.061
24-h mean SBP (per 1 mmHg)	1.0608	0.0157	< 0.001
Office SBP (per 1 mmHg)	-0.027	0.0117	0.021
Sleep duration (per 1 min)	0.0105	0.002	< 0.001
Sleep quality (per 1 point increase)	-0.8093	0.2029	< 0.001

*ABPM* Ambulatory blood pressure monitoring, *eGFR* Estimated glomerular filtration rate, *SBP* Systolic blood pressure

^a^Cardiovascular diseases are defined as a composite of myocardial infarction, coronary artery disease, and stroke

**Table 4:** Multiple linear regression analysis for the factor associated with extent of nighttime systolic blood pressure dipping in hypertensive patients

	β coefficient	Standard error	*p*-value
Age (per 1 year)	-0.0508	0.0174	0.004
Female	1.0285	0.479	0.032
Regular physical activity	-0.2346	0.438	0.592
Body mass index (per 1 kg/m2)	0.0388	0.062	0.532
Current smoking	1.5535	0.6188	0.012
Alcohol drinking	0.7468	0.4793	0.119
Taking antihypertensive drug	0.7231	0.4542	0.112
Medical history of diabetes mellitus	-1.0269	0.529	0.052
Medical history of cardiovascular disease^a^	-0.2341	0.615	0.704
Total cholesterol (per 1 mg/dL)	0.0171	0.005	< 0.001
eGFR (per 1 ml/min/1.73m2)	0.0183	0.0106	0.084
24-h mean SBP (per 1 mmHg)	-0.1171	0.0158	< 0.001
Office SBP (per 1 mmHg)	0.0322	0.0119	0.007
Sleep duration (per 1 min)	-0.0036	0.0021	0.081
Sleep quality (per 1 point increase)	0.7622	0.2052	< 0.001

*ABPM* Ambulatory blood pressure monitoring, *eGFR* Estimated glomerular filtration rate, *SBP* Systolic blood pressure

^a^Cardiovascular diseases are defined as a composite of myocardial infarction, coronary artery disease, and stroke

### Associated factor for nighttime systolic blood pressure dipping less than 10 percent

Table 5 showed the significant associated factors associated with less than 10 percent nighttime SBP which was analyzed using multiple logistic regression analysis with consideration of relevant variables. The result showed that male sex (odds ratio [OR] for female sex = 0.72, 95% confidence interval [CI] = 0.56–0.94), low eGFR (< 60 ml/min/1.73m2) (OR = 1.55, 95% CI = 1.06–2.30), high ambulatory BP (≥ 130/80 mmHg) (OR = 1.76, 95% CI = 1.33–2.33), low office BP (≥ 140/90 mmHg) (OR = 0.56, 95% CI = 0.41–0.75), and poor sleep quality (OR = 0.84, 95% CI = 0.75–0.94) were significant factors associated with nighttime SBP dipping less than 10 percent.

**Table 5:** Multiple logistic regression analysis for the factors associated with nighttime dipping less than 10 percent in hypertensive patients

	Dipping less than 10 percent OR (95% CI)	*p*-value
Age (> 54.5 years)	1.01 (0.99–1.02)	0.127
Female	0.72 (0.56–0.94)	0.014
Regular physical activity	0.99 (0.78–1.25)	0.919
Body mass index (≥ 25 kg/m2)	0.90 (0.72–1.13)	0.379
Current smoking	0.81 (0.58–1.12)	0.200
Alcohol drinking	0.78 (0.60–1.01)	0.057
Taking antihypertensive drug	0.79 (0.62–1.01)	0.064
Medical history of diabetes mellitus	1.19 (0.89–1.59)	0.235
Medical history of dyslipidemia	0.86 (0.67–1.09)	0.216
Medical history of cardiovascular disease^a^	1.19 (0.85–1.67)	0.318
eGFR (< 60 ml/min/1.73m2)	1.55 (1.06–2.30)	0.026
24-h mean BP (≥ 130/80 mmHg)	1.76 (1.33–2.33)	< 0.001
Office BP (≥ 140/90 mmHg)	0.56 (0.41–0.75)	< 0.001
Sleep duration (> 420 min)	1.00 (0.99–1.00)	0.297
Sleep quality (3 or 4 point)	0.84 (0.75–0.94)	0.002

*ABPM* Ambulatory blood pressure monitoring, *OR* Odds ratio, *CI* Confidence interval, *eGFR* Estimated glomerular filtration rate, *BP* Blood pressure

^a^Cardiovascular diseases are defined as a composite of myocardial infarction, coronary artery disease, and stroke

**Fig. 1:**
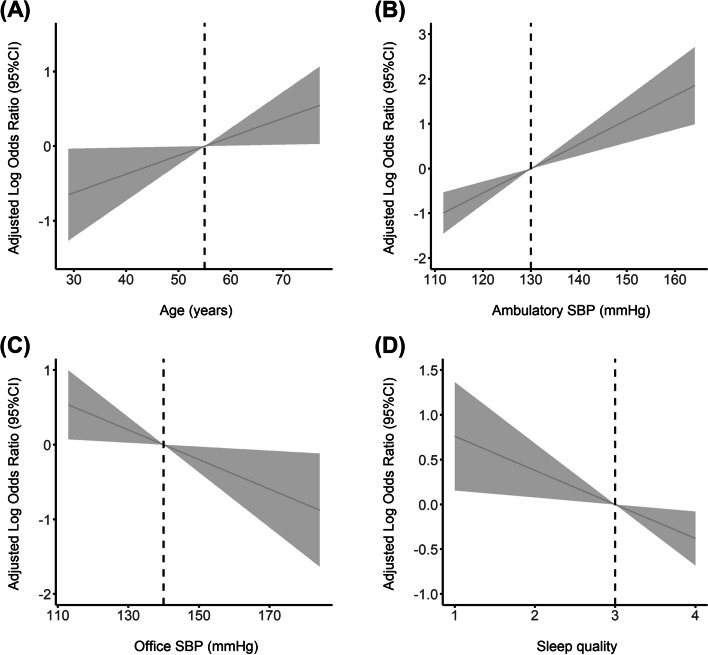
Predictive odds ratios curves for the continuous association between nightthime dipping less than 10% and (**A**) age; (**B**) office systolic blood pressure; (**C**) 24-h mean systolic blood pressure; (**D**) sleep quality by proportional odds ordinal logistic regression models using maximum likelihood estimation. Reference points were 55 year, 140 mmHg, 130 mmHg, and 3 points for age, office systolic blood pressure, 24-h mean systolic blood pressure, and sleep quality, respectively

Figure 1 showed the predictive OR curves for the continuous relationship between age, ambulatory SBP, office SBP, sleep quality and odds of nighttime SBP dipping less than 10 percent. The result showed that age and ambulatory SBP had a positive linear relationship with the odds of nighttime SBP dipping less than 10 percent. On the other hand, office SBP and sleep quality exhibited a negative linear relationship with the odds of nighttime SBP dipping less than 10 percent.

### Associated factor for each nocturnal dipping pattern

Table 6 showed the associated factors for each nocturnal dipping pattern which was analyzed by multiple logistic regression analysis. Taking antihypertensive drugs (OR = 1.91, 95% CI = 1.18–3.09) is the independent factor for extreme dipper. Contrarily, medical history of diabetes mellitus (OR = 0.53, 95% CI = 0.27–0.97) and high ambulatory BP (≥ 130/80 mmHg) (OR = 0.48, 95% CI = 0.29–0.80) are inversely associated with extreme dipper. In addition, low ambulatory BP (OR = 0.68, 95% CI = 0.51–0.90), high office BP (OR = 1.73, 95% CI = 1.27–2.36), and better sleep quality (OR = 1.16, 95% CI = 1.03–1.30) were significant factors associated for dipper.

**Table 6:** Multiple logistic regression analysis for the factors related to each nocturnal dipping pattern in hypertensive patients

	Extreme dipper	Dipper	Non-dipper	Reverse dipper
	OR (95% CI)	OR (95% CI)	OR (95% CI)	OR (95% CI)
Age (> 54.5 years)	0.99 (0.97–1.01)	0.99 (0.98–1.00)	1.00 (0.99–1.01)	1.01 (0.99–1.02)
Female	1.54 (0.91–2.64)	1.25 (0.96–1.63)	0.74 (0.58–0.96)	0.94 (0.65–1.36)
Regular physical activity	0.85 (0.52–1.36)	1.07 (0.84–1.36)	0.89 (0.70–1.12)	1.22 (0.87–1.70)
Body mass index (≥ 25 kg/m2)	1.06 (0.67–1.64)	1.08 (0.86–1.36)	0.92 (0.74–1.16)	0.93 (0.67–1.30)
Current smoking	1.45 (0.81–2.54)	1.09 (0.78–1.52)	0.84 (0.60–1.18)	0.83 (0.47–1.41)
Alcohol drinking	1.18 (0.72–1.95)	1.23 (0.94–1.59)	0.87 (0.67–1.13)	0.79 (0.53–1.16)
Taking antihypertensive drug	1.91 (1.18–3.09)	1.04 (0.81–1.34)	0.81 (0.63–1.03)	0.99 (0.68–1.43)
Medical history of diabetes mellitus	0.53 (0.27–0.97)	0.97 (0.72–1.29)	1.02 (0.77–1.35)	1.29 (0.87–1.89)
Medical history of dyslipidemia	1.15 (0.70–1.84)	1.13 (0.88–1.45)	0.84 (0.66–1.07)	1.06 (0.74–1.50)
Medical history of cardiovascular disease^a^	0.74 (0.36–1.43)	0.94 (0.67–1.34)	1.05 (0.75–1.47)	1.12 (0.72–1.73)
eGFR (< 60 ml/min/1.73m2)	0.40 (0.11–1.00)	0.76 (0.51–1.13)	0.86 (0.59–1.25)	2.30 (1.47–3.54)
24-h mean BP (≥ 130/80 mmHg)	0.48 (0.29–0.80)	0.68 (0.51–0.90)	1.51 (1.15–2.00)	1.31 (0.88–1.97)
Office BP (≥ 140/90 mmHg)	1.32 (0.73–2.37)	1.73 (1.27–2.36)	0.78 (0.58–1.04)	0.55 (0.38–0.80)
Sleep duration (> 420 min)	1.00 (0.99–1.00)	0.99 (0.99–1.00)	1.00 (0.99–1.00)	1.00 (0.99–1.00)
Sleep quality (3 or 4 point)	1.09 (0.87–1.35)	1.16 (1.03–1.30)	0.98 (0.87–1.09)	0.73 (0.62–0.86)

*ABPM* Ambulatory blood pressure monitoring, *OR* Odds ratio, *CI* Confidence interval, *eGFR* Estimated glomerular filtration rate, *BP* Blood pressure

^a^Cardiovascular diseases are defined as a composite of myocardial infarction, coronary artery disease, and stroke

In non-dipper pattern, male sex was significant associated factor (OR for female sex = 0.74, 95% CI = 0.58–0.96). High ambulatory BP (≥ 130/80 mmHg) was also significant associated factor for non-dipper (OR = 1.51, 95% CI = 1.15–2.00), but low office BP (≥ 140/90 mmHg) was not. In addition, low eGFR (< 60 ml/min/1.73m2) (OR = 2.30, 95% CI = 1.47–3.54) and poor sleep quality (OR = 0.73, 95% CI = 0.62–0.86) were significant associated factors for reverse dipper. Contrary to dipper, high office BP (≥ 140/90 mmHg) was significant associated factor for reverse dipper (OR = 1.81, 95% CI = 1.23–2.64), but high ambulatory BP (≥ 130/80 mmHg) was not. Sleep duration was not associated with all of nocturnal dipping pattern.

### Comparison among the different definitions of nighttime BP

BP patterns and comparisons of the proportion of dipping patterns according to different definitions of nighttime BP are summarized in Tables S2 and S3. The extent of nighttime SBP according to the time weighted actual sleep time method, non-time weighted actual sleep time method, narrow fixed interval method, and deep sleep method were 8.81 ± 7.94%, 9.27 ± 8.22%, 8.95 ± 8.97%, and 10.35 ± 9.44%, respectively. When nighttime BP was defined according to non-time weighting method, the proportions of extreme dipper, dipper, non-dipper, and reverse dipper were 8.55%, 37.76%, 45.52%, and 13.55%, respectively. The proportions extreme dipper, dipper, non-dipper, and reverse dipper were 10.26%, 35.00%, 37.37%, and 16.32% in the definition according to the narrow fixed interval method, and 14.34%, 36.71%, 31.32%, and 13.82% in the definition according to the deep sleep method (overall *p*-value = 0.005).

Table S4 showed comparison of factors related to dipping less than 10 percent in hypertensive patients according to the different definitions of nighttime BP. Low eGFR (< 60 ml/min/1.73m2), low office BP (≥ 140/90 mmHg), and poor sleep quality were factors consistently showed significant association with dipping less than 10 percent. Age, sex, and medical history of dyslipidemia showed significant associations according to some definitions of nighttime BPs. Sleep duration was independent associated factor for dipping less than 10 percent only when nighttime BP was defined by non-time weighting method.

## Discussions

In this study, we demonstrated that sleep quality was significantly associated with nighttime SBP and extent of nighttime SBP dipping. Also, better sleep quality was independently associated with dipper and poor sleep quality was independently associated with reverse dipper. In addition, associated factor for nighttime SBP and extent of nighttime SBP dipping were age, sex, SBP, smoking status, and total cholesterol. Furthermore, male sex, low eGFR, high ambulatory BP, low office BP, and poor sleep quality were significant associated factors for nighttime SBP dipping less than 10 percent. It is very well known that non-dipper or reverse dipper is associated with poor cardiovascular prognosis [30, 31], and the association between nighttime dipping and cardiovascular prognosis could be non-linear or J shape as suggested by the association between extreme dipper and cardiovascular prognosis [32]. However, there are few studies reporting the epidemiologic features and associated factor for nighttime dipping and/or the phenotype of nighttime BP pattern [23]. In our study, clinical characteristics and associated factor for nighttime BP and nocturnal dipping pattern were demonstrated in a relatively large-sized study population.

Previous studies reported that age, sleep disorder, alcohol consumption, and various comorbidities such as diabetes mellitus and chronic kidney disease can affect to nighttime BP [11, 12, 18]. In our study, the nighttime SBP dipping was positively associated with younger age, female sex, higher office SBP, smoking, higher total cholesterol, and lower 24-h mean SBP. Younger age, higher office SBP, and smoking can be explained by the increased daytime activity or stress and/or responsiveness to the stress during daytime, which can be relieved by sleep or rest. In particular, smoking showed positive trend with extreme dipper and negative trend with reverse dipper in Table 6. These findings can be explained by the possibility that smoking can increase daytime BP level thus increasing nighttime SBP dipping [33]. Nighttime SBP dipping is defined by 24-h BP, so the association between office BP and nighttime SBP dipping is relatively weak. As shown in Table 4 and Table S1, interpretation should be cautious as the association between office SBP and nighttime SBP dipping may vary depending on whether 24-h mean SBP is adjusted or not.

In our study, the difference between daytime and nighttime SBP was similar between untreated hypertensive patients and treated hypertensive patients, therefore nighttime SBP dipping was similar in both groups. In addition, taking antihypertensive drug was not independent factor for nighttime SBP and extent of nighttime SBP dipping. This finding suggests that antihypertension medication itself does not have effect on improvement of non-dipper or reverse dipper pattern. One interesting finding was that taking antihypertensive drug was associated with extreme dipper pattern. Little is known about the factors associated with extreme dippers, so further studies are needed on this finding.

More than a half of the patients were non-dipper or reverse dipper in our study. This prevalence is much higher than that in the general population. For example, in the Ohasama study population in Japan, the prevalence of non-dipper or reverse dipper was only 16% [34]. However, in a study conducted with subjects had high BP in office, the prevalence of non-dipper or reverse dipper was 41% to 52.8%, which is similar with our results [17]. Previous studies reported that non-dipper was associated with obesity, aging, autonomic dysfunction, and overt cardiovascular and renal disease [17, 35]. However, in our study, these factors did not show significant association with non-dipper or reverse dipper phenotype, except for low eGFR (< 60 ml/min/1.73m2) which showed significant association with reverse dipper.

From a clinical point of view, it is unclear whether non-dipper and reverse dipper are distinct phenotypes. When we combine these two patterns and analyze them in Table 5, male sex, low eGFR, high ambulatory BP, low office BP, and poor sleep quality were independent associated factor for nighttime dipping less than 10 percent. In addition, when analyzed using different definitions of nighttime BP, only low eGFR, low office BP, and poor sleep quality were consistently associated factors for nighttime dipping less than 10 percent. Moreover, low eGFR (< 60 ml/min/1.73m2) was significantly associated with reverse dipper in our study. This finding was consistent with the previous study [36], and may suggest the clinical importance of accompanying chronic kidney disease in hypertensive patients. In our study, age was associated with extent of nighttime SBP dipping, but was not associated with non-dipper or reverse dipper patterns. It is still controversial whether non-dipper is related to age itself or to factors such as sleep quality and arterial stiffness, which increase with aging [37, 38].

In our study, as shown in Table 6, hypertension by office BP and sleep quality are closely associated with both dipper and reverse dipper. This finding is important for the following reasons. First, recent findings suggest that sleep quality is not affected by ABPM and that poor sleep quality could be associated with hypertension itself regardless of the non-dipper or dipper pattern [39]. Second, regarding the poor sleep quality, sleep apnea syndrome has been spotlighted recently [40]. For extreme dipper, for which there is a controversy regarding the clinical implications as a prognostic factor or as a marker for morning surge [41], sleep quality could be an important confounding variable. Nocturnal hypertension has been long known to be associated with cardiovascular prognosis without any breakthrough, and has not been utilized for clinical applications. To address this challenge, our study showed that sleep quality could be another path to understanding the unfavorable patterns of nighttime BP.

There is insufficient evidence to support the preference for any specific definition of sleep time in ABPM [42]. In our study, when nighttime BP is defined by actual sleep time, male sex, low eGFR, high ambulatory BP, low office BP, and poor sleep quality were associated factor blunted nighttime SBP dipping. But the dipping patterns themselves are affected by the different method to define sleep time.

Limitations in our study need to be mentioned. Firstly, the association is only cross-sectional so that the mechanism and/or causality behind the association are unknown. Secondly, our data is registry data. Therefore, laboratory data were collected at the physician’s discretion according to own usual standards of care. However, the participating patient population was large enough to explore the cross-sectional relationship, and the result from multiple linear regression analysis is quite consistent with multiple logistic regression analyses. Thirdly, even though the four-point Likert scale for the questionnaire determining sleep quality is adopted for this study, this scale was not validated using gold standard polysomnography and is therefore quite subjective.

## Conclusions

In conclusion, based on the data from KORABP registry, extent of nighttime SBP dipping was associated with age, sex, SBP, smoking status, total cholesterol, and sleep quality. Moreover, blunted nighttime SBP dipping was associated with male sex, low eGFR, high ambulatory BP, low office BP, and poor sleep quality. In addition, it was found that better sleep quality was associated with dipper and poor sleep quality was associated with reverse dipper.

## Supplementary Information

**Additional file 1: Table S1.** Additional multiple linear regression analysis performed by excluding 24-hour mean SBP and Office SBP for the factor associated with extent of nighttime systolic blood pressure dipping in hypertensive patients. **Table S2. **Blood pressure patterns according to the different definitions of nighttime blood pressure. **Table S3.** Comparisons of the proportion of dipping patterns according to the different definitions of nighttime blood pressure. **Table S4.** Multiple logistic regression analysis for the factors associated with nighttime dipping less than 10 percent in hypertensive patients according to the different definitions of nighttime blood pressure.

## Data Availability

The datasets used and/or analyzed during the current study are available from the corresponding author on reasonable request.

## Abbreviations

BP: Blood pressure
ABPM: Ambulatory blood pressure monitoring
KORABP: Korean Registry for Ambulatory Blood Pressure Monitoring
SBP: Systolic blood pressure
DBP: Diastolic blood pressure
AAMI: Association for the Advancement of Medical Instrumentation
ESH: European Society of Hypertension
eGFR: Estimated glomerular filtration rate
CKD-EPI: Chronic Kidney Disease Epidemiology Collaboration
BMI: Body mass index
OR: Odds ratio
CI: Confidence interval
