# Supplementary material for: Clinical and life style factors related to the nighttime blood pressure, nighttime dipping and their phenotypes in Korean hypertensive patients
Source: Clin Hypertens. 2023 Aug 1;29:21. doi: 10.1186/s40885-023-00241-w (PMC10391961; doi:10.1186/s40885-023-00241-w)
Supplement: Supplementary file 1 — Additional file 1: Table S1. Additional multiple linear regression analysis performed by excluding 24-hour mean SBP and Office SBP for the factor associated with extent of nighttime systolic blood pressure dipping in hypertensive patients. Table S2. Blood pressure patterns according to the different definitions of nighttime blood pressure. Table S3. Comparisons of the proportion of dipping patterns according to the different definitions of nighttime blood pressure. Table S4. Multiple logistic regression analysis for the factors associated with nighttime dipping less than 10 percent in hypertensive patients according to the different definitions of nighttime blood pressure. [file 40885_2023_241_MOESM1_ESM.docx]

| **Table S1**. Additional multiple linear regression analysis performed by excluding 24-hour mean SBP and Office SBP for the factor associated with extent of nighttime systolic blood pressure dipping in hypertensive patients   \|  \| Without 24-hour mean SBP \| \| Without office SBP \| \| \| --- \| --- \| --- \| --- \| --- \| \|  \| β coefficient \| *p*-value \| β coefficient \| *p*-value \| \| Age (per 1 year) \| -0.0511 \| 0.004 \| -0.0510 \| 0.003 \| \| Female \| 1.0329 \| 0.034 \| 1.0224 \| 0.033 \| \| Regular physical activity \| -0.1522 \| 0.732 \| -0.2443 \| 0.576 \| \| Body mass index (per 1 kg/m2) \| -0.0114 \| 0.856 \| 0.0445 \| 0.472 \| \| Current smoking \| 1.3096 \| 0.037 \| 1.5180 \| 0.014 \| \| Alcohol drinking \| 0.5994 \| 0.218 \| 0.7261 \| 0.129 \| \| Taking antihypertensive drug \| 0.8099 \| 0.079 \| 0.6940 \| 0.125 \| \| Medical history of diabetes mellitus \| -0.9924 \| 0.065 \| -1.0058 \| 0.057 \| \| Medical history of cardiovascular disease^*^ \| -0.3620 \| 0.561 \| -0.4787 \| 0.433 \| \| Total cholesterol (per 1 mg/dL) \| 0.0179 \| <0.001 \| 0.0180 \| <0.001 \| \| eGFR (per 1 ml/min/1.73m2) \| 0.0194 \| 0.072 \| 0.0165 \| 0.120 \| \| 24-hour mean SBP (per 1 mmHg) \|  \|  \| -0.0968 \| <0.001 \| \| Office SBP (per 1 mmHg) \| -0.0101 \| 0.341 \|  \|  \| \| Sleep duration (per 1 min) \| -0.0027 \| 0.199 \| -0.0032 \| 0.121 \| \| Sleep quality (per 1 point increase) \| 0.8574 \| <0.001 \| 0.7986 \| <0.001 \| |
| --- | --- | --- | --- | --- | --- | --- | --- | --- | --- | --- | --- | --- | --- | --- | --- | --- | --- | --- | --- | --- | --- | --- | --- | --- | --- | --- | --- | --- | --- | --- | --- | --- | --- | --- | --- | --- | --- | --- | --- | --- | --- | --- | --- | --- | --- | --- | --- | --- | --- | --- | --- | --- | --- | --- | --- | --- | --- | --- | --- | --- | --- | --- | --- | --- | --- | --- | --- | --- | --- | --- | --- | --- | --- | --- | --- | --- | --- | --- | --- | --- | --- | --- | --- | --- | --- |

ABPM, ambulatory blood pressure monitoring; eGFR, estimated glomerular filtration rate; SBP, systolic blood pressure;

^*^Cardiovascular diseases are defined as a composite of myocardial infarction, coronary artery disease, and stroke.

| **Table S2.** Blood pressure patterns according to the different definitions of nighttime blood pressure | | | | | |
| --- | --- | --- | --- | --- | --- |
|  | ^*^Time weighted  actual sleep time  method | ^†^Non-time weighted  actual sleep time method | ^§^Narrow fixed interval method | ^¶^Deep sleep method |  |
| 24-hour mean SBP (mmHg) | 135.92±15.28 | 137.49±15.15 | 136.99±15.32 | 136.46±15.08 |  |
| 24-hour mean DBP (mmHg) | 85.15±10.82 | 86.28±10.87 | 85.96±11.08 | 85.57±10.92 |  |
| Daytime mean SBP (mmHg) | 139.94±15.50 | 140.43±15.38 | 140.77±15.78 | 140.77±15.78 |  |
| Daytime mean DBP (mmHg) | 88.12±11.39 | 88.44±11.35 | 88.71±11.73 | 88.71±11.73 |  |
| Nighttime mean SBP (mmHg) | 127.53±17.64 | 127.32±17.72 | 127.97±17.70 | 125.97±17.77 |  |
| Nighttime mean DBP (mmHg) | 78.93±11.48 | 78.85±11.47 | 79.28±11.61 | 77.93±11.57 |  |
| Nighttime SBP dipping (%) | 8.81±7.94 | 9.27±8.22 | 8.95±8.97 | 10.35±9.44 |  |
| Nocturnal dipping pattern |  |  |  |  |  |
| Extreme dipper | 7.63% | 8.55% | 10.26% | 14.34% |  |
| 24-hour mean SBP (mmHg) | 130.71±12.40 | 134.22±12.69 | 135.40±13.75 | 135.73±14.81 |  |
| 24-hour mean DBP (mmHg) | 82.41±10.18 | 85.38±10.66 | 85.97±10.15 | 85.77±10.78 |  |
| Daytime mean SBP (mmHg) | 142.01±13.59 | 141.82±13.50 | 145.92±14.86 | 146.01±15.73 |  |
| Daytime mean DBP (mmHg) | 89.36±11.80 | 90.15±11.61 | 92.45±10.99 | 92.09±11.74 |  |
| Nighttime mean SBP (mmHg) | 108.63±10.90 | 107.66±10.96 | 110.81±12.07 | 110.06±12.89 |  |
| Nighttime mean DBP (mmHg) | 68.65±7.92 | 68.48±8.09 | 70.71±9.04 | 69.84±8.95 |  |
| Nighttime SBP dipping (%) | 23.49±2.68 | 24.08±2.99 | 24.03±3.43 | 24.61±3.66 |  |
| Dipper | 36.45% | 37.76% | 35.00% | 36.71% |  |
| 24-hour mean SBP (mmHg) | 133.50±14.08 | 136.43±14.44 | 136.29±14.84 | 135.21±13.64 |  |
| 24-hour mean DBP (mmHg) | 84.23±10.18 | 86.12±10.59 | 85.78±11.25 | 85.54±10.78 |  |
| Daytime mean SBP (mmHg) | 139.88±14.82 | 140.96±14.87 | 142.40±15.23 | 141.28±14.15 |  |
| Daytime mean DBP (mmHg) | 88.66±10.81 | 89.24±11.09 | 90.09±11.75 | 89.88±11.47 |  |
| Nighttime mean SBP (mmHg) | 119.80±12.94 | 120.39±12.92 | 121.67±13.69 | 120.60±12.61 |  |
| Nighttime mean DBP (mmHg) | 74.76±9.44 | 75.19±9.58 | 75.49±10.12 | 75.21±9.94 |  |
| Nighttime SBP dipping (%) | 14.33±2.68 | 14.57±2.78 | 14.56±2.84 | 14.62±2.84 |  |
| Non-dipper | 42.76% | 45.52% | 37.37% | 31.32% |  |
| 24-hour mean SBP (mmHg) | 137.69±14.53 | 138.53±14.44 | 137.20±14.20 | 137.13±14.68 |  |
| 24-hour mean DBP (mmHg) | 86.32±11.05 | 86.85±11.02 | 86.45±10.85 | 85.73±10.71 |  |
| Daytime mean SBP (mmHg) | 140.21±14.92 | 140.38±14.69 | 139.53±14.46 | 139.51±15.05 |  |
| Daytime mean DBP (mmHg) | 88.35±11.57 | 88.35±11.38 | 88.25±11.29 | 87.61±11.12 |  |
| Nighttime mean SBP (mmHg) | 132.35±14.18 | 132.33±14.06 | 131.64±13.90 | 131.59±14.10 |  |
| Nighttime mean DBP (mmHg) | 82.01±10.72 | 81.85±10.63 | 82.05±10.55 | 81.19±10.51 |  |
| Nighttime SBP dipping (%) | 5.57±2.66 | 5.71±2.66 | 5.63±2.73 | 5.63±2.81 |  |
| Reverse dipper | 13.16% | 13.55% | 16.32% | 13.82% |  |
| 24-hour mean SBP (mmHg) | 139.86±20.00 | 139.45±19.59 | 139.02±19.25 | 139.01±19.18 |  |
| 24-hour mean DBP (mmHg) | 85.48±11.77 | 85.59±11.39 | 85.24±11.83 | 85.04±12.02 |  |
| Daytime mean SBP (mmHg) | 138.02±19.73 | 138.21±19.35 | 137.12±19.06 | 137.08±18.98 |  |
| Daytime mean DBP (mmHg) | 85.11±11.84 | 85.37±11.43 | 84.69±11.90 | 84.58±12.08 |  |
| Nighttime mean SBP (mmHg) | 144.20±21.41 | 144.22±21.42 | 143.88±20.34 | 144.00±20.57 |  |
| Nighttime mean DBP (mmHg) | 86.44±12.27 | 86.73±12.20 | 86.44±12.15 | 86.20±12.60 |  |
| Nighttime SBP dipping (%) | -4.47±3.48 | -4.30±3.54 | -4.98±3.95 | -5.09±4.24 |  |

Data are presented as mean ± standard deviation. SBP, systolic blood pressure; DBP, diastolic blood pressure; BP, blood pressure.

^*^Average BP defined using time weighted actual sleep time method was calculated as nighttime BP x actual sleeping duration/24 + daytime BP x (24 minus actual sleep duration)/24.

^†^Average BP defined using non-time weighted actual sleep time method was calculated based on actual sleep time without weighting.

^§^Nighttime BP by narrow fixed interval method was defined by average BP from midnight to 5AM.

^¶^Nighttime BP during deep sleep method was defined by the average BP between the time of 2 hours after sleep and the time of 1 hour before awakening.

| **Table S3**. Comparisons of the proportion of dipping patterns according to the different definitions of nighttime blood pressure.   \|  \| Extreme dipper \| Dipper \| Non-dipper \| Reverse dipper \| \| --- \| --- \| --- \| --- \| --- \| \| Time weighted actual sleep time method (A) \| 7.63% \| 36.45% \| 42.76% \| 13.16% \| \| Non-time weighted actual sleep time method (B) \| 8.55% \| 37.76% \| 45.52% \| 13.55% \| \| Narrow fixed interval method (C) \| 10.26% \| 35.00% \| 37.37% \| 16.32% \| \| Deep sleep method (D) \| 14.34% \| 36.71% \| 31.32% \| 13.82% \| \| *p*-value differences \|  \|  \|  \|  \| \| 4 by 4^*^ \| 0.005 \| \| \| \| \| 1 by 4^*^ \| <0.001 \| 0.235 \| <0.001 \| <0.001 \| \| (A) vs (B) ^†^ \| 0.121 \| 0.194 \| 0.014 \| 0.700 \| \| (A) vs (C) ^†^ \| 0.001 \| 0.403 \| 0.002 \| <0.001 \| \| (A) vs (D) ^†^ \| <0.001 \| 0.743 \| <0.001 \| 0.082 \| \| (B) vs (C) ^†^ \| 0.049 \| 0.105 \| 0.152 \| 0.001 \| \| (B) vs (D) ^†^ \| <0.001 \| 0.934 \| <0.001 \| 0.233 \| \| (C) vs (D) ^†^ \| <0.001 \| 0.121 \| <0.001 \| 0.049 \| |
| --- | --- | --- | --- | --- | --- | --- | --- | --- | --- | --- | --- | --- | --- | --- | --- | --- | --- | --- | --- | --- | --- | --- | --- | --- | --- | --- | --- | --- | --- | --- | --- | --- | --- | --- | --- | --- | --- | --- | --- | --- | --- | --- | --- | --- | --- | --- | --- | --- | --- | --- | --- | --- | --- | --- | --- | --- | --- | --- | --- | --- | --- | --- | --- | --- | --- | --- | --- | --- | --- | --- |

BP, blood pressure.

^*^Cochran's Q test was used to compare the proportions of more than two paired samples.

^†^McNemar’s test was used to compare the proportions of each dipping pattern.

| **Table S4**. Multiple logistic regression analysis for the factors associated with nighttime dipping less than 10 percent in hypertensive patients according to the different definitions of nighttime blood pressure   \|  \| Odds ratios (95% confidence interval) for dipping less than 10 percent \| \| \| \| --- \| --- \| --- \| --- \| \|  \| Non-time weighted  actual sleep time method \| Narrow fixed interval method \| Deep sleep method \| \| Age (>54.5 years) \| 1.01 (1.00-1.02) \| 1.01 (0.99-1.02) \| 1.02 (1.01-1.03) \| \| Female \| 0.82 (0.63-1.06) \| 0.73 (0.56-0.95) \| 0.73 (0.56-0.95) \| \| Regular physical activity \| 0.92 (0.73-1.17) \| 0.88 (0.69-1.11) \| 0.96 (0.75-1.21) \| \| Body mass index (≥25 kg/m2) \| 1.00 (0.80-1.26) \| 0.98 (0.78-1.23) \| 1.00 (0.79-1.25) \| \| Current smoking \| 0.79 (0.56-1.11) \| 0.93 (0.66-1.30) \| 0.90 (0.63-1.26) \| \| Alcohol drinking \| 0.79 (0.61-1.03) \| 0.97 (0.75-1.25) \| 0.88 (0.68-1.14) \| \| Taking antihypertensive drug \| 0.81 (0.63-1.04) \| 0.99 (0.77-1.27) \| 0.93 (0.72-1.19) \| \| Medical history of diabetes mellitus \| 1.17 (0.87-1.56) \| 1.17 (0.88-1.56) \| 1.07 (0.80-1.43) \| \| Medical history of dyslipidemia \| 0.82 (0.64-1.05) \| 0.78 (0.61-0.99) \| 0.79 (0.62-1.01) \| \| Medical history of cardiovascular disease^*^ \| 1.16 (0.83-1.63) \| 1.20 (0.86-1.69) \| 1.34 (0.96-1.89) \| \| eGFR (<60 ml/min/1.73m2) \| 1.48 (1.01-2.17) \| 1.55 (1.06-2.29) \| 1.61 (1.1-2.37) \| \| 24-hour mean BP (≥130/80 mmHg) \| 1.26 (0.93-1.70) \| 1.20 (0.90-1.61) \| 1.21 (0.91-1.62) \| \| Office BP (≥140/90 mmHg) \| 0.63 (1.47-0.85) \| 0.64 (0.47-0.86) \| 0.63 (0.47-0.84) \| \| Sleep duration (>420 min) \| 1.00 (1.00-1.00) \| 1.00 (0.99-1.00) \| 1.00 (0.99-1.00) \| \| Sleep quality (3 or 4 point) \| 0.81 (0.73-0.91) \| 0.84 (0.75-0.93) \| 0.83 (0.74-0.93) \| |
| --- | --- | --- | --- | --- | --- | --- | --- | --- | --- | --- | --- | --- | --- | --- | --- | --- | --- | --- | --- | --- | --- | --- | --- | --- | --- | --- | --- | --- | --- | --- | --- | --- | --- | --- | --- | --- | --- | --- | --- | --- | --- | --- | --- | --- | --- | --- | --- | --- | --- | --- | --- | --- | --- | --- | --- | --- | --- | --- | --- | --- | --- | --- | --- | --- | --- | --- | --- | --- |

eGFR, estimated glomerular filtration rate; BP, blood pressure.

^*^Cardiovascular diseases are defined as a composite of myocardial infarction, coronary artery disease, and stroke.
